# Comparison of problem-based learning and traditional teaching methods in medical psychology education in China: A systematic review and meta-analysis

**DOI:** 10.1371/journal.pone.0243897

**Published:** 2020-12-14

**Authors:** Junwei Gao, Ling Yang, Jinghui Zhao, Lian Wang, Jiao Zou, Chunxiang Wang, Xiaotang Fan

**Affiliations:** Department of Developmental Neuropsychology, School of Psychology, Third Military Medical University (Army Medical University), Chongqing, China; University of Eastern Finland, SWEDEN

## Abstract

**Background:**

PBL approach has been widely used in many Chinese universities over the past decade. However, the effects of PBL approach on medical psychology education in China are inconsistent. The purpose of this study was to ascertain whether the PBL approach was superior to the lecture-based teaching method in the context of the medical psychology curriculum in China.

**Methods:**

A systematic review and meta-analysis was performed to confirm the effectiveness of PBL in Chinese medical psychology. Corresponding databases were searched for available studies, where data were extracted to calculated Hedges’ g and its 95% confidence interval in total and subgroup analyses. Subgroup analyses were also carried out.

**Results:**

Nine studies with 551 cases and 496 controls were identified. The total examination scores of students in the PBL approach group were significantly higher compared with students in the traditional lecture-based teaching group under the random effect model (Hedges’ g = 1.510, 95%CI 0.792–2.227, p<0.001). Subgroup analyses based on major and school system exhibited similar results.

**Conclusions:**

Our study supported the notion that the PBL approach may be applicable to Chinese medical psychology education.

## Introduction

Problem-based learning (PBL) is an educational approach, originally introduced by Barrows and Tamblyn in the 1960s [1, 2]. It is characterized by the application of problems for knowledge acquisition and practical skills [3, 4]. The PBL approach is student-centered compared with the traditional teaching model. Students are encouraged to participate in the discussion of prepared problems to improve themselves in the process [5, 6]. The PBL approach aims to promote the integration of learned knowledge, rather than simply implanting knowledge and skills [7]. Therefore, this teaching model has been highly praised in medical education courses in the past two decades, and medical psychology has been studied extensively.

Medical psychology is an emerging discipline in medical universities worldwide and plays a bridge role between basic and clinical courses in medical education [8]. With the transformation of the medical model from a single biomedical model to a multidisciplinary bio-psycho-social model, increasing attention has been paid to medical psychology. Medical students are said to acquire basic psychological knowledge and to develop improved skills in dealing with the doctor-patient relationship through their learning in this course. However, medical psychology teaching faces some unique difficulties compared with other basic and medicine courses. For instance, there is no specific specimen or model for intuitive display teaching. Medical students must rely only on their wisdom and imagination to understand corresponding problems. At present, Chinese medical colleges universally lag behind Western colleges in their medical psychology teaching content, style, and methodology [9]. Some researchers tried to introduce the PBL approach into medical psychology coursework in Chinese medical colleges and universities to promote teaching reform and accelerate improvement in teaching outcomes [10]. However, the results were controversial. A previous study suggested that the PBL approach did not significantly affect students’ final exam scores of medical psychology [11]. Therefore, we conducted the systematic review and meta-analysis to verify whether the PBL approach has a positive effect on medical psychology education in China.

## Materials and methods

### Publication search

We searched all records in the PubMed, Web of Knowledge, Embase, China National Knowledge Infrastructure (CNKI), and Wan-fang databases with no language restrictions up to March 13, 2020. The combination of terms was as follows: ((problem-based learning) OR PBL) AND (medical psychology). The references of retrieved articles were also checked to avoid missing additional studies.

### Inclusion and exclusion criteria

The inclusion criteria were as follows: (1) Research subjects were students in Chinese colleges or universities; (2) Studies evaluated the effect of the PBL approach on medical psychology education; (3) Students’ final exam scores for medical psychology were presented as a mean with a corresponding standard deviation or sample size and p value in the full text. The exclusion criteria were as follows: (1) Studies with insufficient data; (2) Reviews, comments, or abstracts. For studies with overlapping data, only the one with the largest sample size was included. When an article reported results on different groups, we treated them as separate studies.

### Data extraction

Two authors (JG and LY) independently identified records and screened corresponding titles, abstracts and full texts. Data were extracted into a predesigned Excel sheet by two reviewers (JG and LY). The extracted data were collected from the included studies as follows: the first author’s name, publication year, source and major of students, school system, proportion of PBL teaching time, educational approach, outcome measures, duration of course, sample size, mean and standard deviation (SD) of medical psychology scores in both groups, or p value from the studies. Discrepancies were settled through discussion with a third person (JZ). Attempts were also made to contact authors for missing data.

### Quality assessment

The methodological quality of studies was evaluated by two independent reviewers (JG and LY) according to the Newcastle–Ottawa Scale (NOS), which contains three dimensions: selection, comparability, and exposure or outcome. Eight items were included to assess the quality of studies. Disagreements were resolved by discussion with a third author (JZ).

### Statistical analysis

Effect sizes (standardized mean differences, SMDs) were calculated according to sample size and mean (SD) values or sample size and P values. Then, we converted the SMD to Hedges’ g, which serves a more unbiased role. Data were then pooled together using Hedges’ g as appropriate. A fixed effect model or random effect model was chosen depending on heterogeneity analysis results, which were assessed by the I^2^ value and Chi-square based Q-test. When the effect was evaluated to be heterogeneous, a random effect model was selected; otherwise, a fixed effect model was used. In addition to the overall analysis, subgroup analyses were also conducted according to the major and school system of students. A Galbraith plot was drawn to explore the source of between-study heterogeneity. Sensitivity analysis was performed by removing each study sequentially. Publication bias was assessed by the Begg’s test. All statistical tests were performed using Comprehensive Meta-analysis 2.0 software (Biostat Inc) and STATA 12.0 (STATA Corporation).

## Results

### Study characteristics

**Fig 1:**
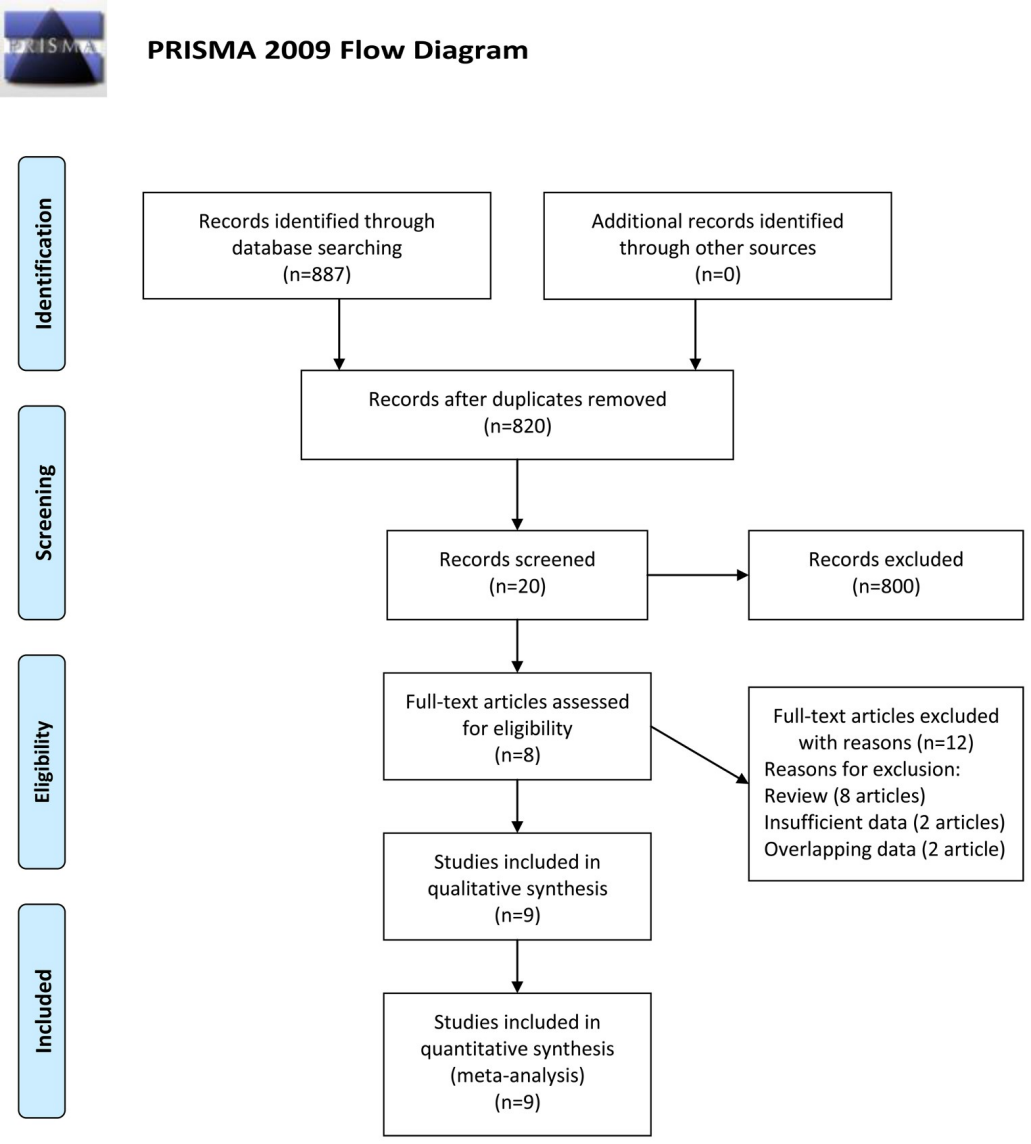
Flow diagram of study identification.

There were 887 records identified in the initial search up to March 13, 2020. Sixty-seven duplicate records were found among these results. Scanning of titles and abstracts help to remove 800 records. Further, several studies were excluded by full-text reading because of insufficient data (2 articles), overlapping data (2 articles) and review (8 articles). We treated the record by Dang et al. [12], which reported results on different ethnicities, as two separate studies. Ultimately, eight records (nine studies) with 551 cases and 496 controls were included in our meta-analysis [11–18] (Flow diagram in Fig 1).

Of the nine studies, the majority of participants were in clinical medicine. The duration of a medical psychology course was generally one semester. The sample sizes ranged from 11 to 114 students in PBL groups and control groups. Most studies not only included the final examination results of both groups but also carried out a corresponding questionnaire, whose specific items varied in the included studies. Generally, students were satisfied with the PBL approach. Nearly half of the studies were conducted with medical students in the five-year system. The results of methodological quality assessment were shown were shown in Table 1.

**Table 1:** Characteristics of included studies in this meta-analysis.

**Fist author**	**Source of students**	**Major of students**	**School system**	**Proportion of**	**Outcome measures**
**Publication year**				**PBL teaching time**	
Yi 2007	Qingdao University Medical College	Clinical medicine	Five-year system	20–30%	Theoretical scores/satisfaction survey
Pan 2009	West China Medical College	Clinical medicine	Five-year system	50%	Theoretical scores
Zhang 2011	Yichun University	Clinical medicine	Five-year system	18 class hours	Theoretical scores
Long 2012	Guangzhou Medical College	Medical imaging and Health law	Not described	Not described	Theoretical scores/satisfaction survey
Huang 2014	Yongzhou Vocational and Technical College	Clinical medicine	Three-year system	100%	Theoretical scores/satisfaction survey
Wang 2017	Capital Medical University	Clinical medicine	Five-year system	100%	Theoretical scores/satisfaction survey
Wang 2019	Air Force Medical University	Clinical medicine	Five-year system	100%	Theoretical scores/satisfaction survey
Dang (a) 2014	Xinjiang Medical University	Clinical and Preventive medicine	Not described	44.4%	Theoretical scores/satisfaction survey
Dang (b) 2014	Xinjiang Medical University	Clinical and Preventive medicine	Not described	44.4%	Theoretical scores/satisfaction survey
**Duration of course**	**PBL group**		**Control group**		**Methodological quality**
	**Students (n)**	**Total scores (Mean±SD)**	**Students (n)**	**Total scores (Mean±SD)**	
One semester	114	85.70±8.96	114	81.10±8.76	6
One semester	11	81.55±4.11	11	75.45±3.67	7
One semester	48	85.00±8.49	48	72.00±8.06	6
One semester	99	12.56±4.86	38	12.15±3.58	6
One semester	58	85.00±2.70	58	72.00±2.80	6
Half of the semester	60	89.92±1.76	60	83.18±3.41	7
One semester	36	88.2.0±5.40	36	77.20±6.30	7
One semester	64	0.001^a^	66		7
One semester	61	0.001^a^	65		7

^a^ data shown as sample size and p value.

### Quantitative data synthesis

**Fig 2:**
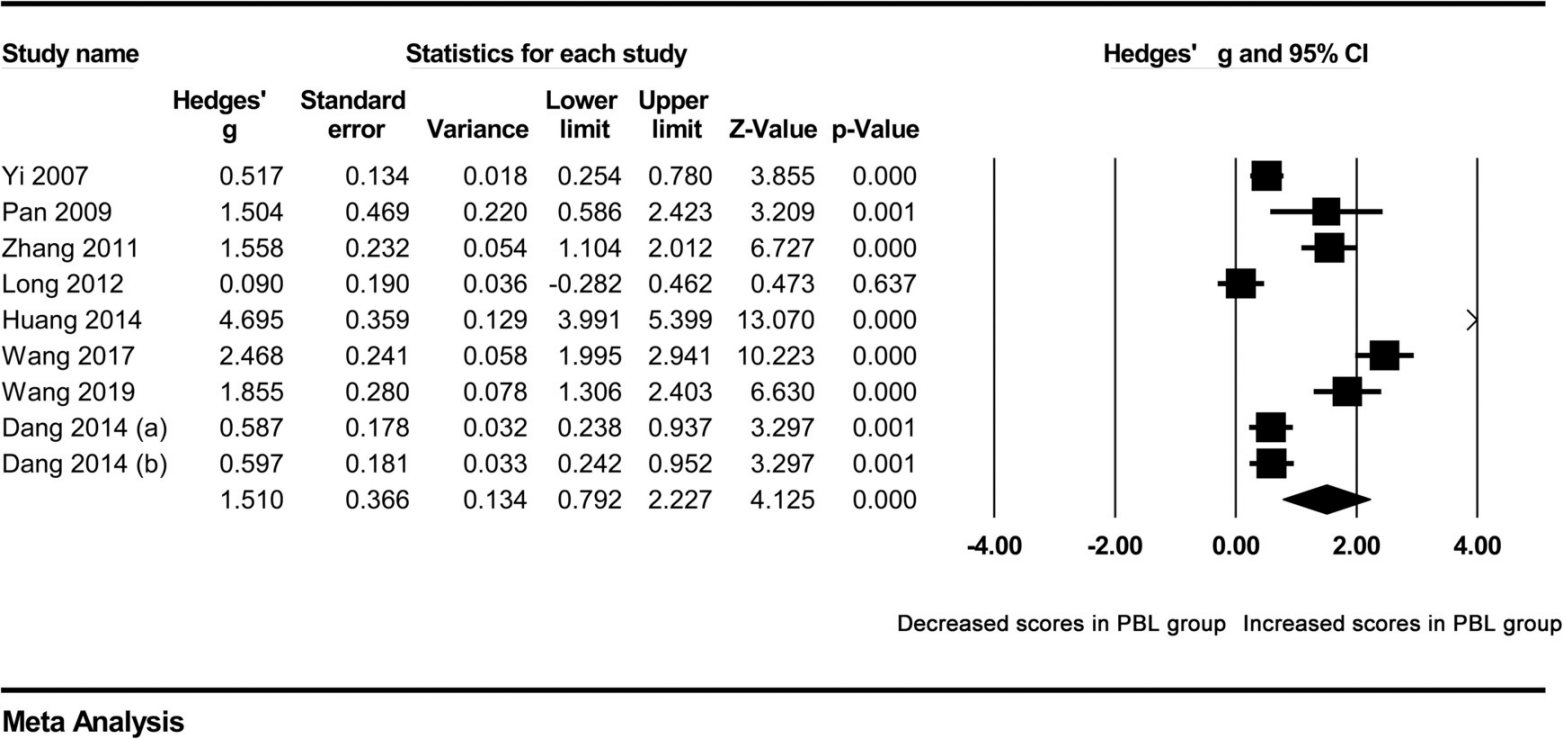
Forest plot for the random-effect meta-analysis.

In our meta-analysis, the combined results of the overall comparison revealed a significant difference in the total examination scores of students in favor of the PBL approach, compared with traditional teaching under the random effect model (Hedges’ g = 1.510, 95%CI 0.792–2.227, p<0.001) (Fig 2) (Table 2).

**Table 2:** Summary of meta-analysis results.

`					Tests of association			Tests of heterogeneity			Publication bias
Groups	Studies (n)	Case (n)	Control (n)	Model	Hedges’ g [95%CI]	Z	P value	Q value	p value	I^2^ (%)	Begg’s p value
Overall	9	551	496	RE	1.510 [0.792–2.227]	4.125	<0.001	205.309	<0.001	96.103	0.048
Clinical medicine	6	327	327	RE	2.086 [0.997–3.174]	3.754	<0.001	149.966	<0.001	96.666	0.348
Five-year system	5	269	269	RE	1.571 [0.751–2.391]	3.755	<0.001	61.738	<0.001	93.521	0.807
Removing studies outside the boundaries in Galbraith plot	4	250	256	FE	0.592 [0.415–0.770]	6.543	<0.001	4.099	0.251	26.807	

RE random effects model, FE fixed effects model.

### Investigation of heterogeneity

**Fig 3:**
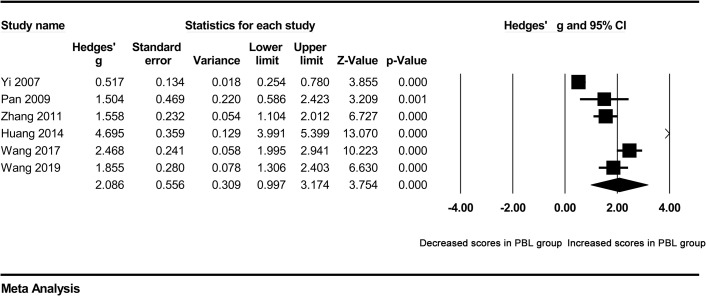
Forest plot for the random-effect meta-analysis of the clinical medicine subgroup.

**Fig 4:**
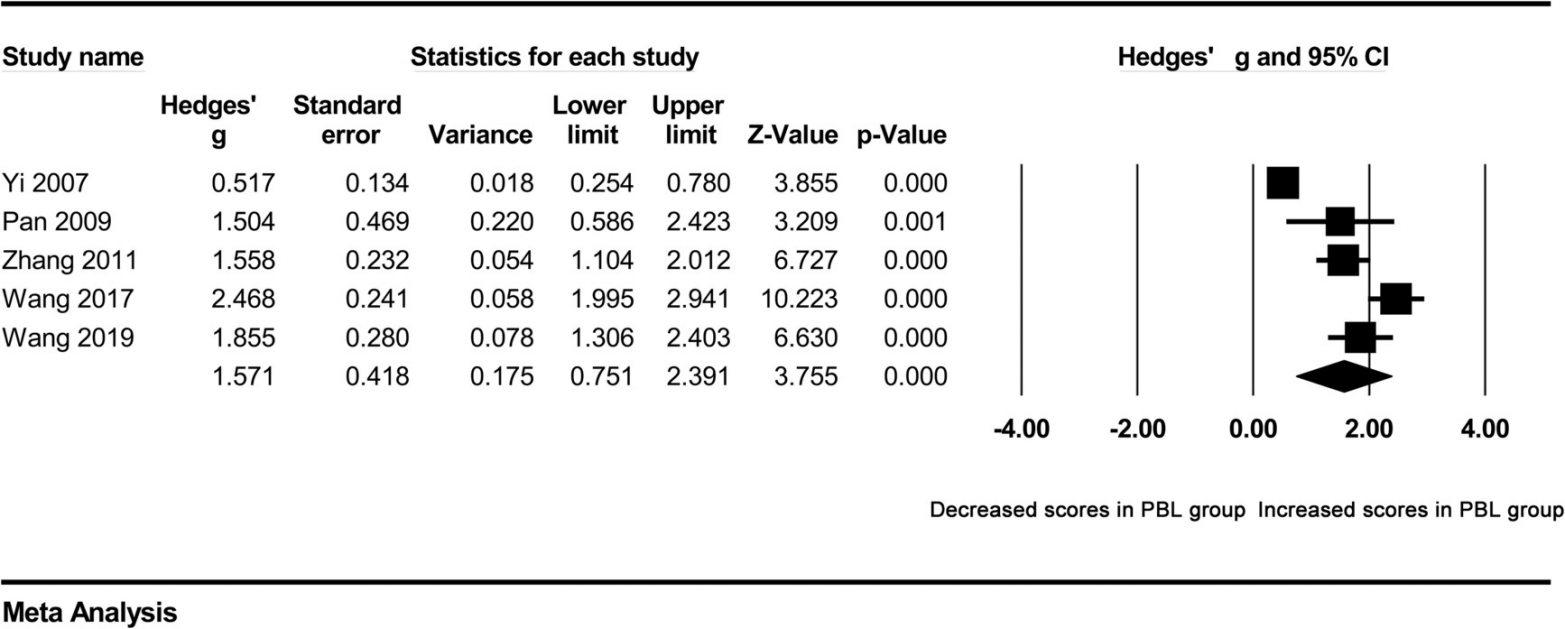
Forest plot for the random-effect meta-analysis of the five-year system subgroup.

**Fig 5:**
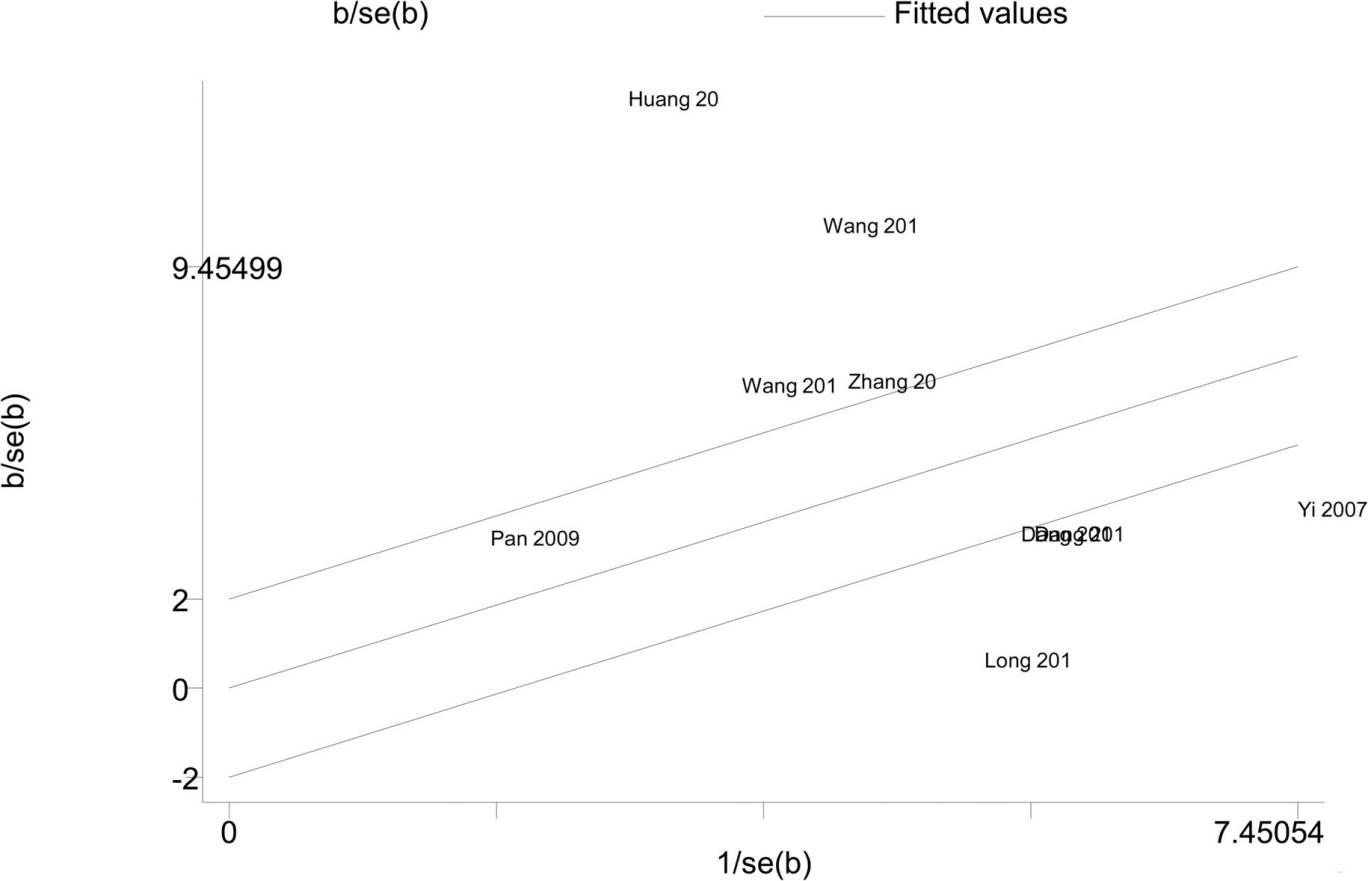
Galbraith plot for the random-effect meta-analysis.

**Fig 6:**
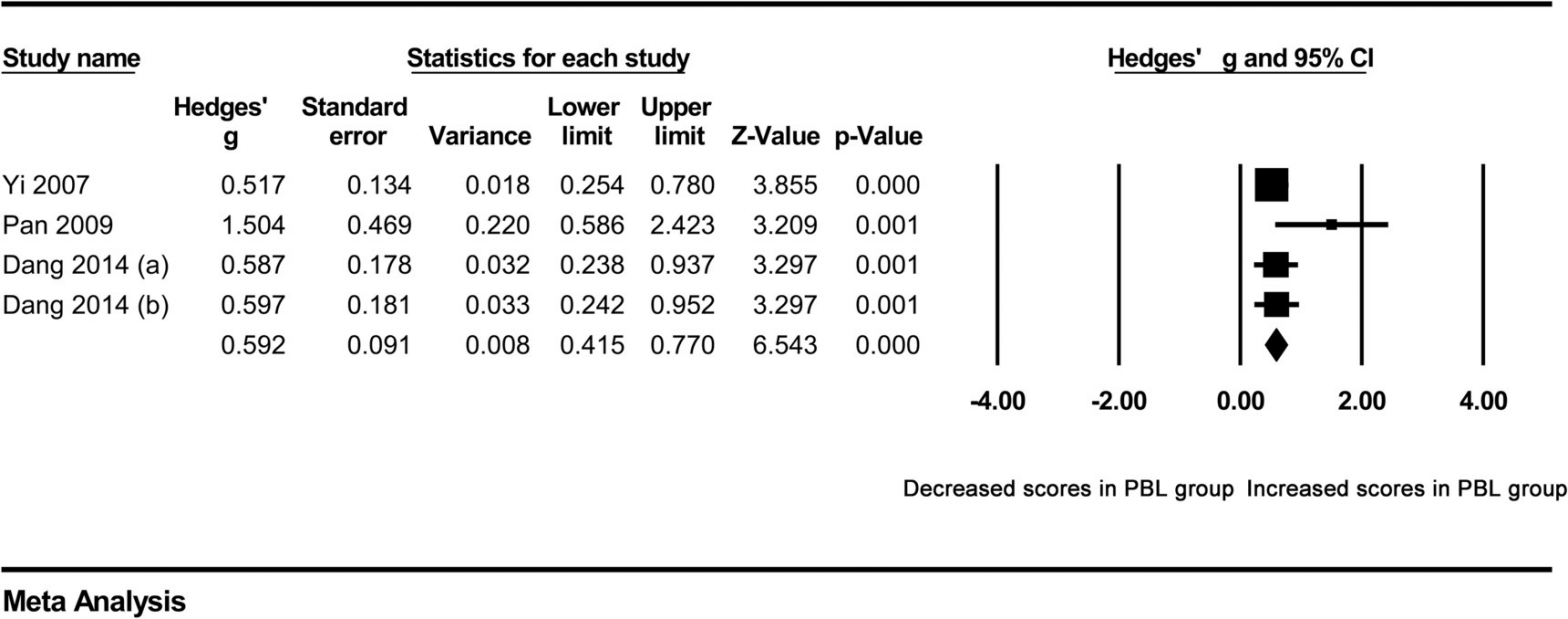
Forest plot for the fixed-effect meta-analysis after removing the studies outside the boundaries of the Galbraith plot.

A high degree of heterogeneity across studies was detected in overall comparisons (P<0.001), which was why we selected the random effect model. Next, we attempted to identify the source of heterogeneity. Subgroup analyses were carried out according to the major and school system of students. The total examination scores of clinical medicine students were significantly higher in the PBL group than in the control group (Hedges’ g = 2.086, 95% CI 0.997–3.174, p<0.001) (Fig 3). Moreover, the total examination scores of five-year system students were still significantly higher in the PBL group than in the control group (Hedges’ g = 1.571, 95% CI 0.751–2.391, p<0.001) (Fig 4). However, heterogeneity was not obviously decreased. In addition, a Galbraith plot was drawn to identify some studies that were obviously different from others, which suggested that studies conducted by Zhang 2011, Long et al. 2012, Huang 2014, Wang et al. 2019, and Wang et al. 2017 [11, 15–18] may affect the results of heterogeneity analysis (Fig 5). After removing them, no obvious heterogeneity was found across studies (I^2^ = 26.807%, p = 0.251). A forest plot indicated that the total examination scores of students in the PBL group were still significantly higher than those of controls (Hedges’ g = 0.592, 95% CI 0.415–0.770, p<0.001) (Table 2) (Fig 6).

### Sensitivity analysis

**Fig 7:**
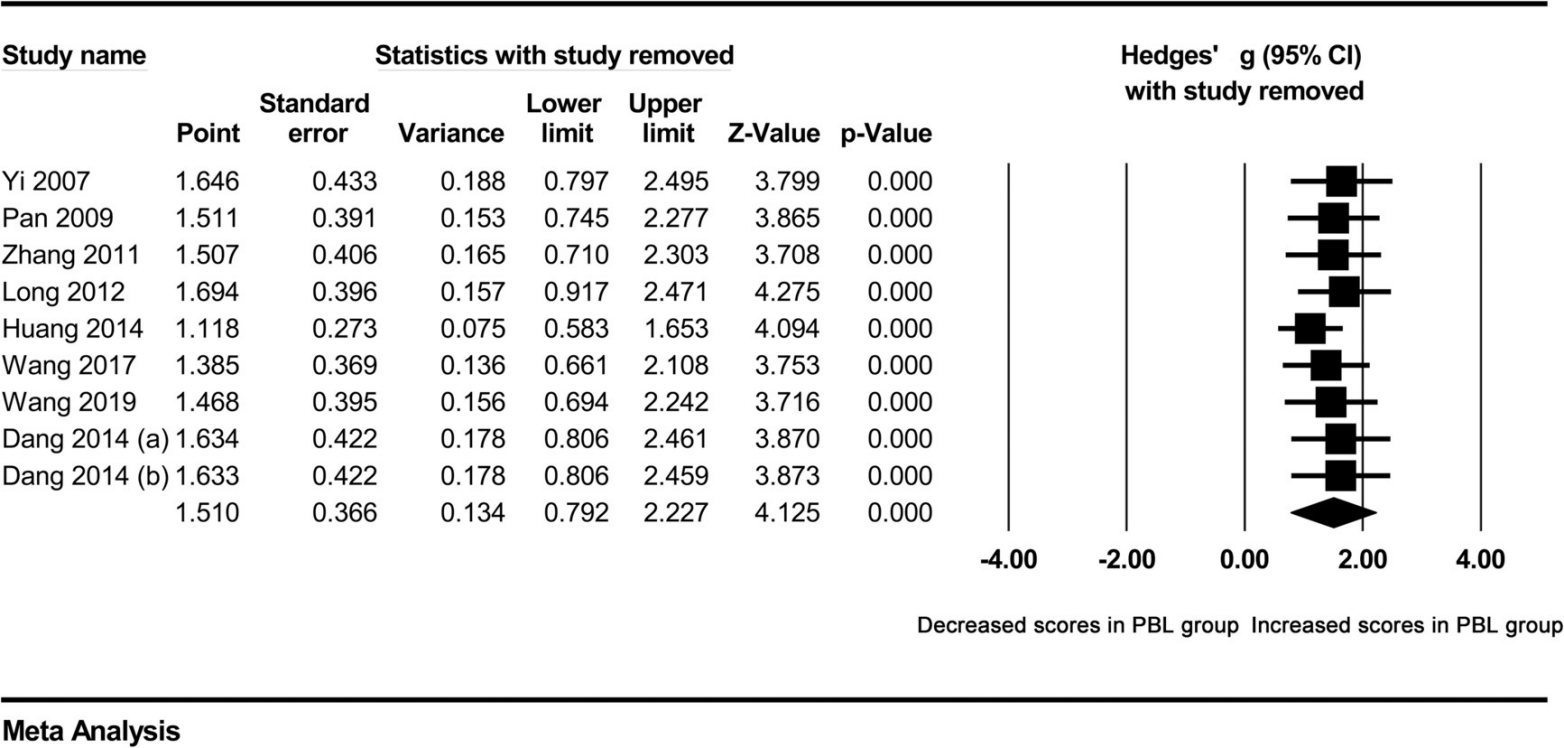
Sensitivity analysis.

Sensitivity analysis was conducted to access the influence of each individual study on pooled Hedges’ g. The results showed that no single study could affect the statistically significant difference in total examination scores between students in PBL groups and controls (Fig 7).

### Publication bias

**Fig 8:**
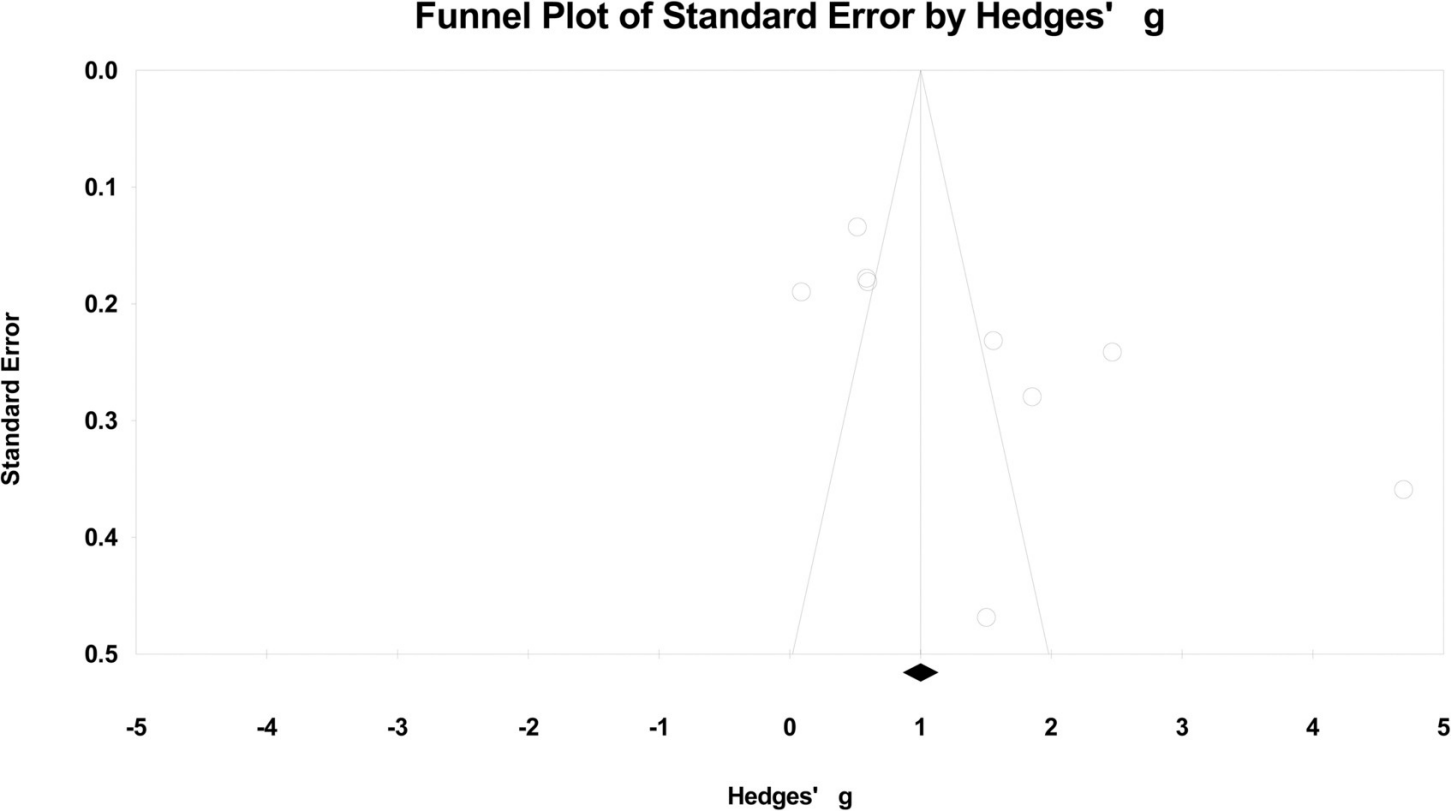
Funnel plot of precision using Hedges’ g statistics.

The shape of the funnel plot was slightly asymmetrical (Fig 8), the result of Begg’s test indicated the p value is around the critical value (p = 0.048). To investigate whether publication bias could affect our results, publication bias was also evaluated in subgroup analyses. No publication bias was found in subgroups, and the association changed little.

## Discussion

Although many efforts have been made in medical education reform, it is still critical that college educators design, develop and implement a series of innovative medicine curriculums, which could guide undergraduate medical students to strive to become doctors [19]. As a bridge course between basic medicine and clinical medicine, medical psychology provides medical students with psychological knowledge and helps them establish the bio-psycho-social concept of illness for future clinical work [20]. In recent years, educators have tried to introduce the PBL approach into Chinese medical psychology classes. Compared with the traditional teaching method, PBL has several advantages in the developed world. Students were encouraged to take responsibility for their own learning, which was an essential skill in their professional development. The PBL approach changed the traditional relationships between teaching and learning. Students elaborated their subjective initiative rather than simply listening and taking notes [21, 22]. Considering the students’ cultural background and social development, some previous studies showed that it was still controversial whether the PBL approach was superior to traditional teaching methods for use in medical psychology classes at Chinese colleges and universities. Therefore, we performed the present meta-analysis to determine the effect of the PBL approach on students’ total scores in a final medical psychology examination in China.

To the best of our knowledge, this is the first meta-analysis to investigate whether the PBL approach is superior to the traditional lecture-based teaching method in Chinese medical psychology course. The strength of this study was that our meta-analysis included more studies with larger sample sizes and showed that the total scores of the PBL group were significantly higher than those of the control group. These results were in line with many previous studies [23–25]. The results of a sensitivity analysis revealed that the correlation was not affected by a particular study, or that it even changed in direction. Moreover, we also noted that high levels of heterogeneity across studies were found in the meta-analysis. Some factors that may cause heterogeneity should be pointed out. First, although these students were all from China, some basic characteristics, such as age, gender, were missing. Second, the teachers and teaching emphasis may be inconsistent in different medical colleges. Third, the proportions of PBL teaching time were different in the included studies. Fourth, the duration of medical psychology course was imprecise. All of these factors may lead to significant heterogeneity in our study. However, for the limited information of the included studies, we could only explore the sources of heterogeneity as much as possible. Subgroup analyses based on school system and major failed to decrease heterogeneity. A Galbraith plot helped us identify five studies that may have been the source of heterogeneity. After removing them, heterogeneity obviously decreased and the correlation did not change. In addition, the results showed that publication bias did not affect our outcomes despite the p value around the critical value. All of these illustrated the credibility of our results. Therefore, we verified that the total examination scores of the PBL group were significantly higher than those of the control group in China. In fact, many medical students and teachers could not accept this teaching mode well, which may be related to the traditional Chinese culture, and most courses were still taught in the traditional way. However, the traditional teaching mode impacted by information technology faced new challenges. Based on such a reality, we suggested trying PBL approach in more courses, so as to verify the results.

Some limitations of our study should be pointed out. First, the sample size was relatively small both in the randomized comparison study and in the meta-analysis, which may potentially have some influence on the results. The homogeneity of the included samples could not be assessed for the limited information, which may be the cause of heterogeneity. Second, the duration of the medical psychology course was relatively short, generally one semester. We could not evaluate the efficacy of PBL in Chinese medical psychology education over a longer period of time. Third, the test to assess the effect of the PBL approach was only performed at the end of the semester, which prevents us from performing delayed assessment for the limited information. Fourth, all of the included studies were performed in China. Whether the conclusion was applicable to other countries and regions was still investigated further. Finally, we did not take cultural factors into account for the limited information. Further studies may focus on this.

## Conclusions

In conclusion, the present study suggested that the PBL approach in medical psychology education in China appeared to be more effective than the traditional teaching method in improving students’ knowledge. Large and well-designed studies are warranted to further confirm whether the PBL approach is superior to different teaching methods in China.

## Supporting information

S1 File PRISMA 2009 checklist. (DOC)
